# Author Correction: Rewiring of the ubiquitinated proteome determines ageing in *C. elegans*

**DOI:** 10.1038/s41586-024-07168-8

**Published:** 2024-02-12

**Authors:** Seda Koyuncu, Rute Loureiro, Hyun Ju Lee, Prerana Wagle, Marcus Krueger, David Vilchez

**Affiliations:** 1 Cologne Excellence Cluster for Cellular Stress Responses in Aging-Associated Diseases (CECAD), University of Cologne, Cologne, Germany; 2 Center for Molecular Medicine Cologne (CMMC), University of Cologne, Cologne, Germany; 3 Faculty of Medicine, University Hospital Cologne, Cologne, Germany

**Keywords:** Proteasome, Ageing

Correction to: *Nature* 10.1038/s41586-021-03781-z Published 28 July 2021

In the version of the article initially published, in the “Western blot” section of the Methods, the sentence “For assessing ubiquitinated proteins by western blot, worms were lysed in lysis buffer (50 mM Tris-HCl, pH 7.8, 150 mM NaCl, 1% Triton X100…” was previously missing “1% Triton X100”. Additionally, in Fig. 1f, “eat-2 D15/WT D15” and “daf-2 D15/WT D15” previously read “eat-2 D15/WT D5” and “daf-2 D15/WT D5”, respectively. These corrections have been made in the HTML and PDF versions of the article.

