# Supplementary material for: Rewiring of the ubiquitinated proteome determines ageing in C. elegans
Source: Nature. 2021 Jul 28;596(7871):285–90. doi: 10.1038/s41586-021-03781-z (PMC8357631; doi:10.1038/s41586-021-03781-z)
Supplement: Supplementary file 1 — This file contains source data for gel electrophoresis. [file 41586_2021_3781_MOESM1_ESM.pdf]

nature portfolio

https://doi.org/10.1038/s41586-021-03781-z

Supplementary information

Rewiring of the ubiquitinated proteome determines ageing in *C. elegans*

In the format provided by the authors and unedited

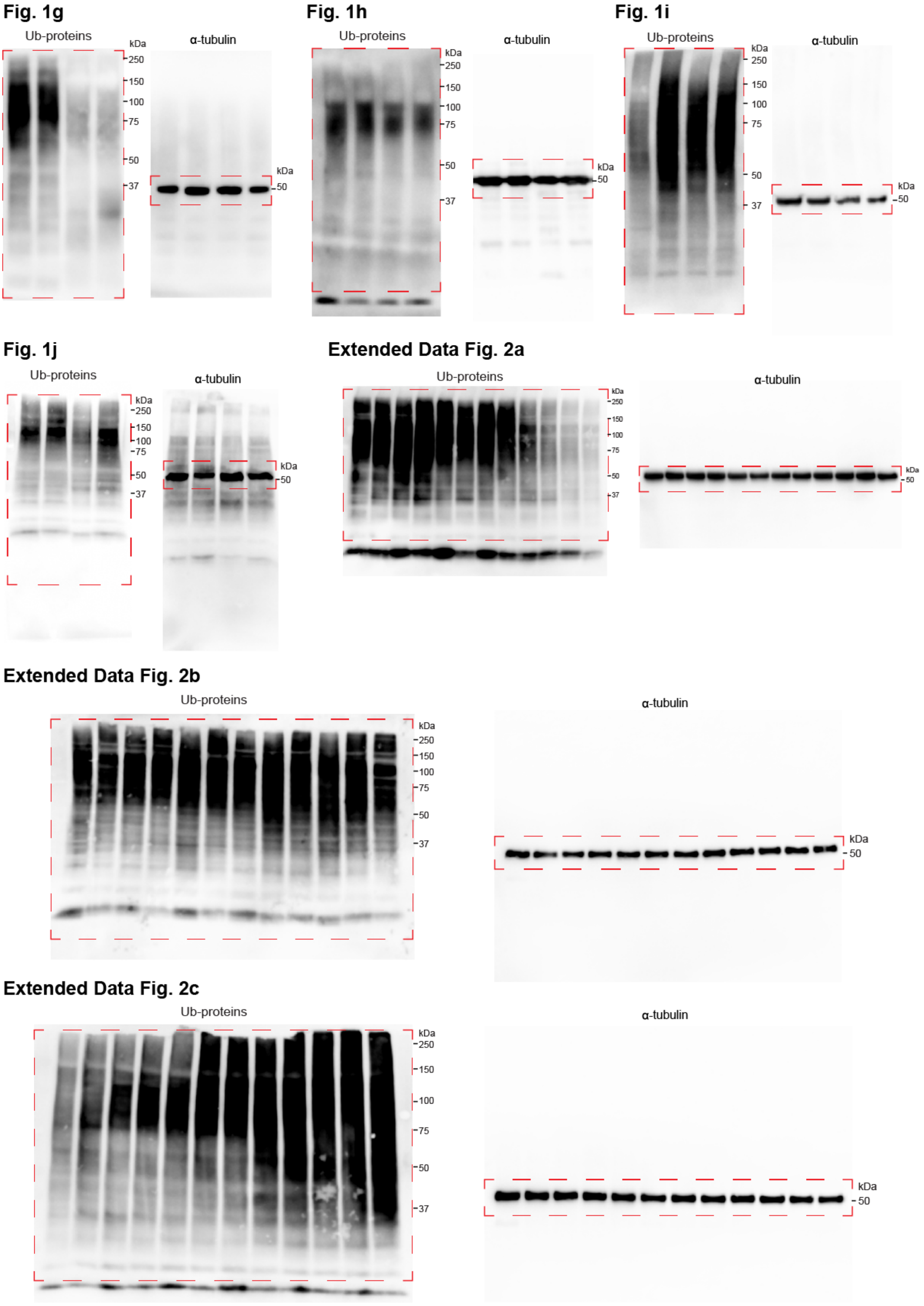

Supplementary Figure 1. Uncropped images are presented with molecular weight ladders.

Extended Data Fig. 2i

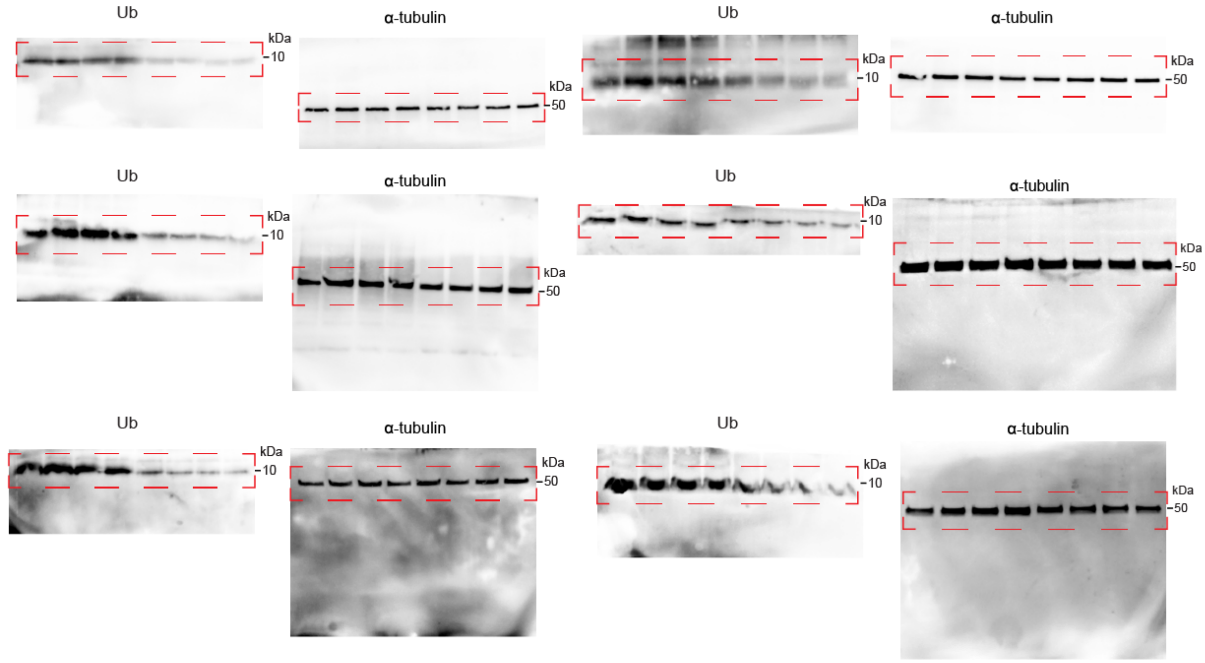

Extended Data Fig. 3c

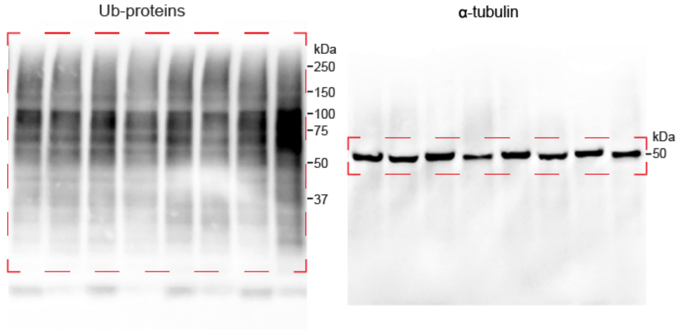

Extended Data Fig. 3d

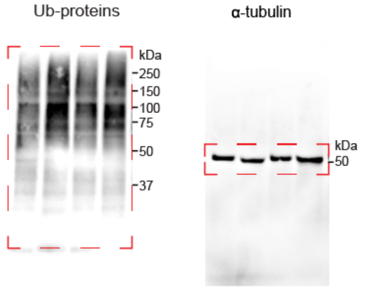

Extended Data Fig. 3e

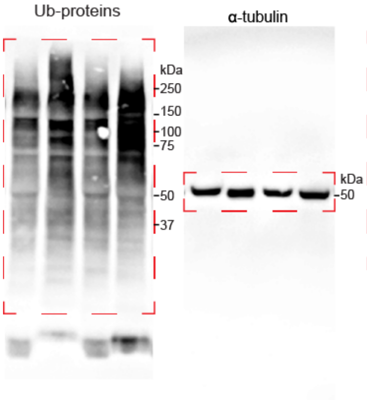

Extended Data Fig. 3f

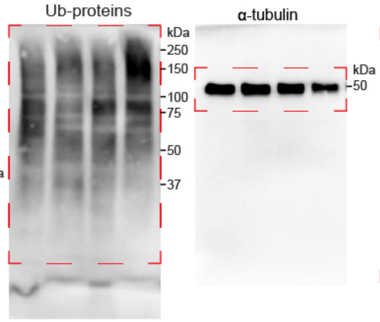

Extended Data Fig. 3g

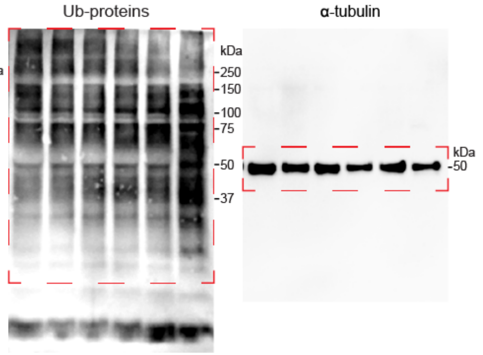

Supplementary Figure 1 (continuation). Uncropped images are presented with molecular weight ladders.

Fig. 2e

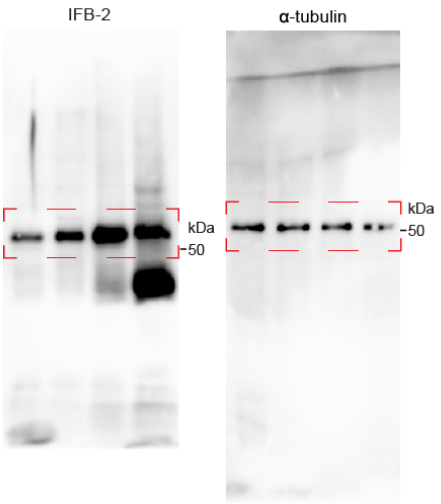

Fig. 2f

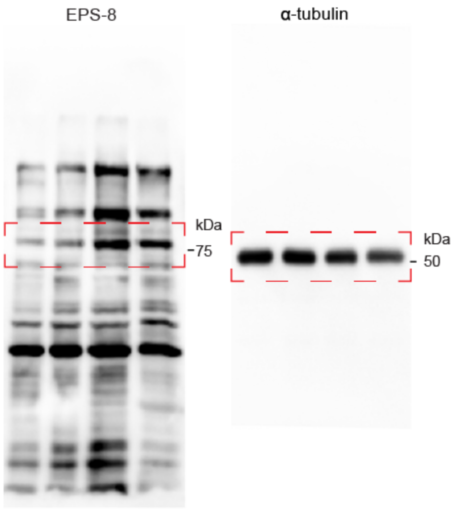

Fig. 2g

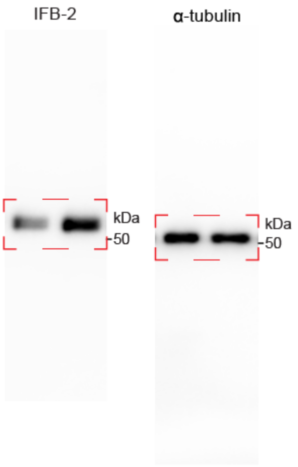

Fig. 2i

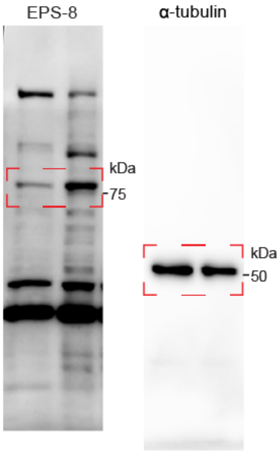

Extended Data Fig. 4f

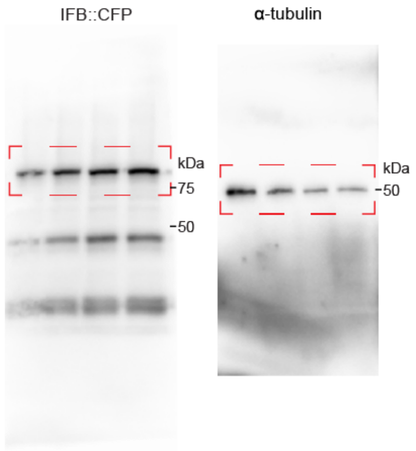

Extended Data Fig. 4g

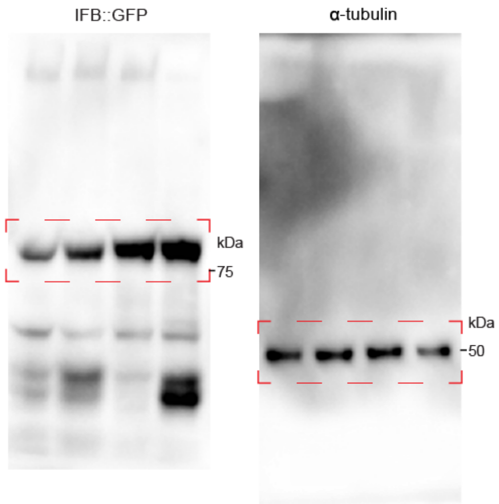

Extended Data Fig. 4h

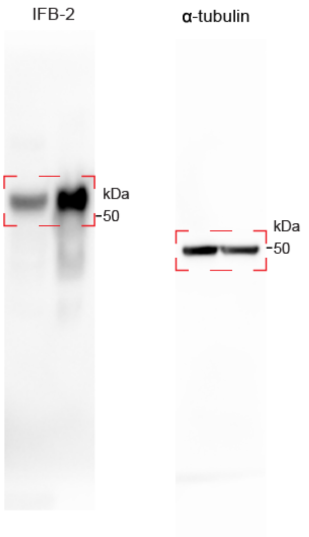

Supplementary Figure 1 (continuation). Uncropped images are presented with molecular weight ladders.

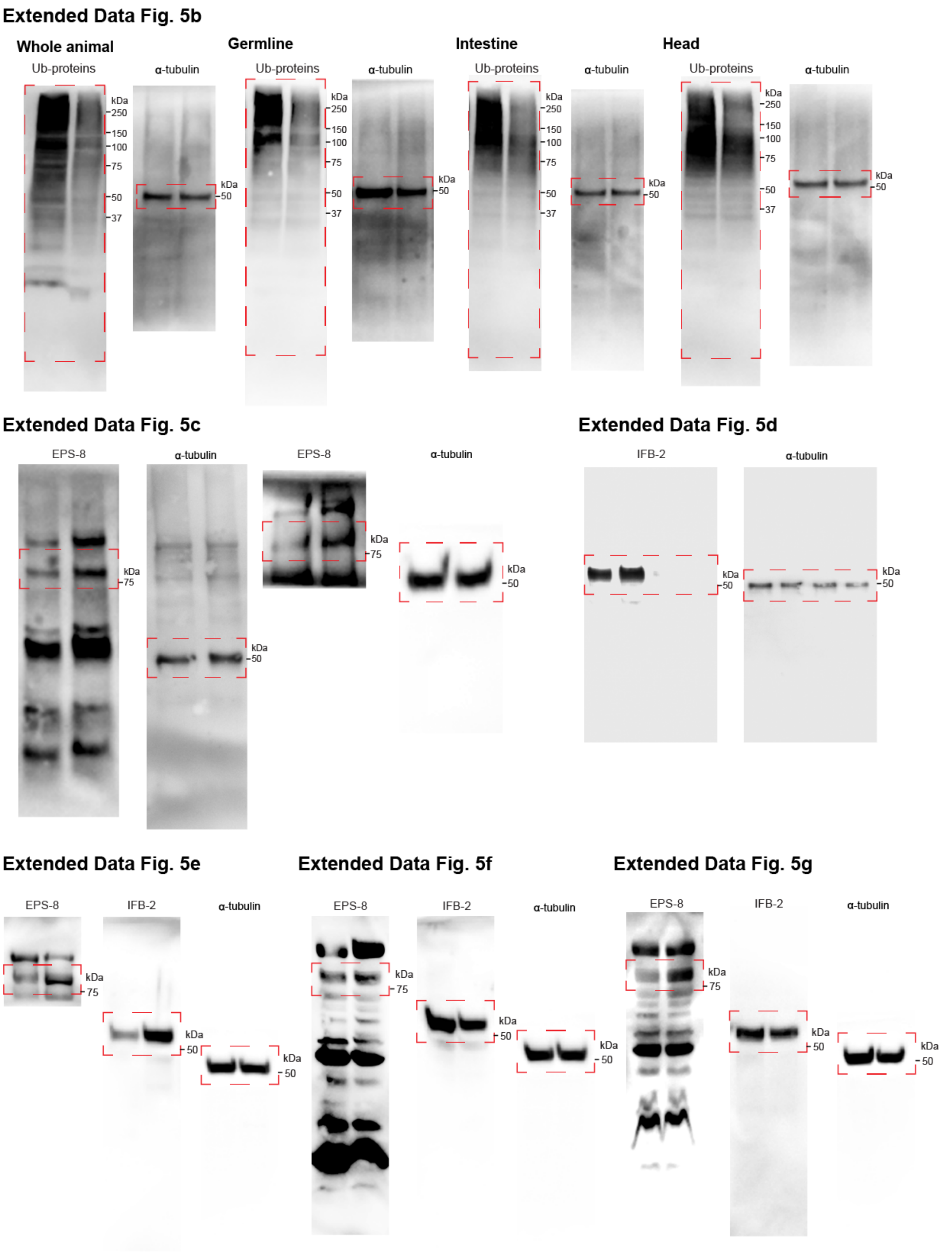

Supplementary Figure 1 (continuation). Uncropped images are presented with molecular weight ladders.

Extended Data Fig. 5h

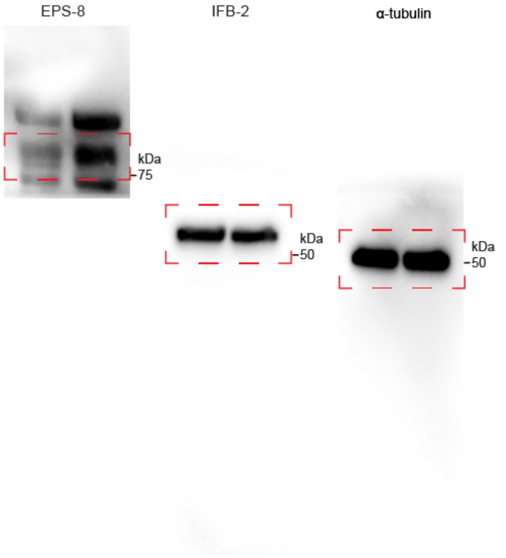

Extended Data Fig. 5i

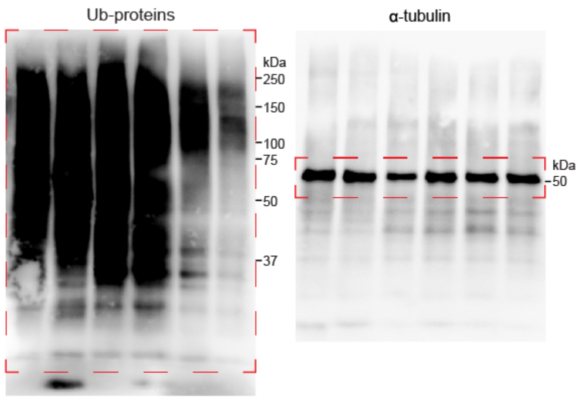

Extended Data Fig. 5j

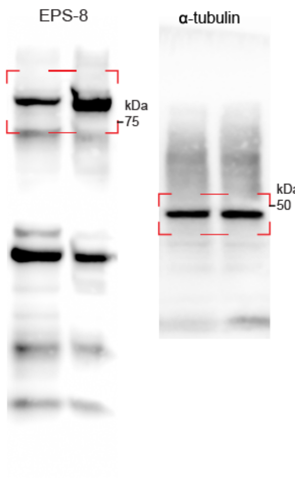

Extended Data Fig. 5k

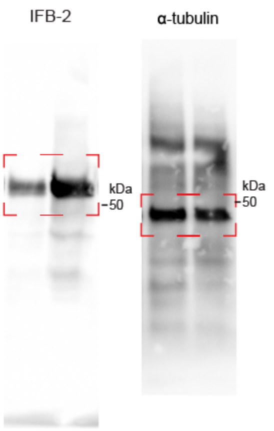

Extended Data Fig. 6a

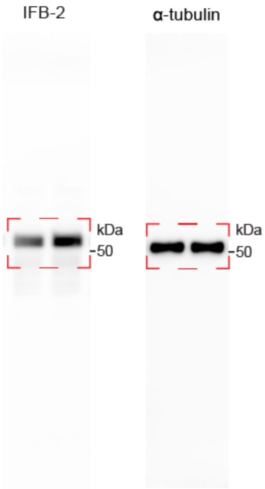

Extended Data Fig. 9g

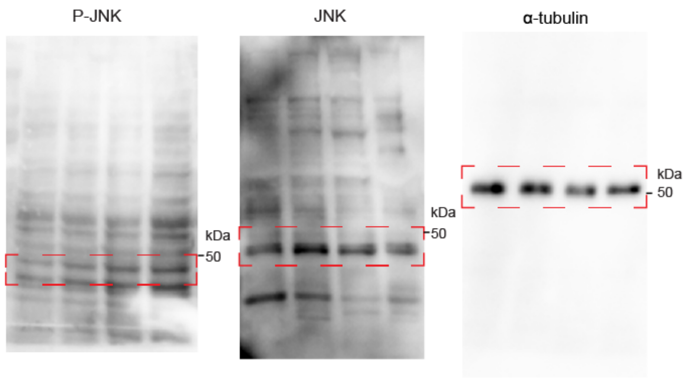

Extended Data Fig. 9h

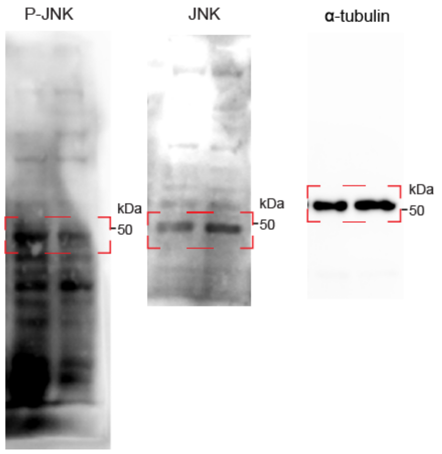

Supplementary Figure 1 (continuation). Uncropped images are presented with molecular weight ladders.
