# Supplementary material for: Rewiring of the ubiquitinated proteome determines ageing in C. elegans
Source: Nature. 2021 Jul 28;596(7871):285–90. doi: 10.1038/s41586-021-03781-z (PMC8357631; doi:10.1038/s41586-021-03781-z)
Supplement: Supplementary file 2 — Reporting Summary [file 41586_2021_3781_MOESM2_ESM.pdf]

nature research

Corresponding author(s): David Vilchez

Last updated by author(s): Jun 21, 2021

Reporting Summary

Nature Research wishes to improve the reproducibility of the work that we publish. This form provides structure for consistency and transparency in reporting. For further information on Nature Research policies, see our Editorial Policies and the Editorial Policy Checklist.

Statistics

For all statistical analyses, confirm that the following items are present in the figure legend, table legend, main text, or Methods section.

n/a	Confirmed	
☐	☒	The exact sample size (*n*) for each experimental group/condition, given as a discrete number and unit of measurement
☐	☒	A statement on whether measurements were taken from distinct samples or whether the same sample was measured repeatedly
☐	☒	The statistical test(s) used AND whether they are one- or two-sided<br>*Only common tests should be described solely by name; describe more complex techniques in the Methods section.*
☒	☐	A description of all covariates tested
☐	☒	A description of any assumptions or corrections, such as tests of normality and adjustment for multiple comparisons
☐	☒	A full description of the statistical parameters including central tendency (e.g. means) or other basic estimates (e.g. regression coefficient) AND variation (e.g. standard deviation) or associated estimates of uncertainty (e.g. confidence intervals)
☐	☒	For null hypothesis testing, the test statistic (e.g. *F*, *t*, *r*) with confidence intervals, effect sizes, degrees of freedom and *P* value noted<br>*Give P values as exact values whenever suitable.*
☒	☐	For Bayesian analysis, information on the choice of priors and Markov chain Monte Carlo settings
☒	☐	For hierarchical and complex designs, identification of the appropriate level for tests and full reporting of outcomes
☒	☐	Estimates of effect sizes (e.g. Cohen's *d*, Pearson's *r*), indicating how they were calculated

Our web collection on statistics for biologists contains articles on many of the points above.

Software and code

Policy information about availability of computer code

Data collection	No software was used
Data analysis	We used GraphPad PRISM 6 for statistical analysis. GraphPad PRISM 6 software was also used to determine median lifespan and generate lifespan graphs. OASIS software (version 1) was used for statistical analysis to determine mean lifespan. For quantification of mCherry fluorescence signal, animals were outlined and quantified using ImageJ software (version 1.51s). For protein identification and label-free quantification (LFQ) in ubiquitin-proteomics experiments, we used the LFQ mode and MaxQuant (version 1.5.3.8) default settings. For proteomics datasets of total protein levels in aging and proteasome-less worms, we used Spectronaut 11 (Biognoys) with the BGS Factory Settings and MaxQuant (version 1.5.3.8) with default settings, respectively.<br><br>All the downstream analyses of the resulting output were performed with R program (version 4.0.5) and Perseus (version 1.6.2.3). Custom code used in this article can be accessed at https://github.com/Vilchezlab/UbProteomics2021. For tissue expression analysis, we used the website http://worm.princeton.edu10.

For manuscripts utilizing custom algorithms or software that are central to the research but not yet described in published literature, software must be made available to editors and reviewers. We strongly encourage code deposition in a community repository (e.g. GitHub). See the Nature Research guidelines for submitting code & software for further information.

Data

Policy information about availability of data

All manuscripts must include a data availability statement. This statement should provide the following information, where applicable:

- Accession codes, unique identifiers, or web links for publicly available datasets
- A list of figures that have associated raw data
- A description of any restrictions on data availability

There is no restriction on data availability. Source data are provided with this paper. Readers can interact with the ubiquitin and global proteomics data using the following Shiny Web apps by downloading the datasets provided in the apps: https://vilchezlab.shinyapps.io/shiny-volcanoplot/ and https://vilchezlab.shinyapps.io/shiny-heatmap/. All the proteomics data have been deposited to the ProteomeXchange Consortium via the PRIDE partner repository with the dataset identifiers PXD024338 (ubiquitin proteomics of aging and long-lived worms), PXD025128 (global protein proteomics of aging and long-lived worms), PXD024094 (ubiquitin proteomics upon rpn-6 RNAi), PXD024095 (global protein proteomics upon rpn-6 RNAi), PXD024093 (immunoprecipitation Lys48-linked polyUb), and PXD024045 (immunoprecipitation Lys63-linked polyUb). MS2 spectra in proteomics experiments were searched against the C. elegans Uniprot database (https://www.uniprot.org/proteomes/UP000001940). For worm tissue expression analysis, we used the database http://worm.princeton.edu.

Field-specific reporting

Please select the one below that is the best fit for your research. If you are not sure, read the appropriate sections before making your selection.

☒ Life sciences ☐ Behavioural & social sciences ☐ Ecological, evolutionary & environmental sciences

For a reference copy of the document with all sections, see nature.com/documents/nr-reporting-summary-flat.pdf

Life sciences study design

All studies must disclose on these points even when the disclosure is negative.

Sample size

No statistical methods were used to predetermine sample size. Exact sample sizes are indicated in the corresponding figure legends.
For ubiquitin and total proteomics experiments, sample sizes were chosen based on prior studies on ubiquitin proteomics in mammalian cells (Kim W et al, Molecular Cell 44, 325-340 (2011)), global proteomics changes during aging in C. elegans (Narayan V et al, Cell Systems 3, 144-159 (2016); Walther D.M. et al, Cell 161, 919-932 (2015)) and our previous work on proteomics analysis of C. elegans (Lee H.J. et al, Nature Metabolism 1, 790-810 (2019)).
Sample sizes for filter traps, western blot, qPCR, motility, lifespan and quantification of fluorescence reporters in C. elegans were determined according to our extensive laboratory experience and other studies using these assays (Lee H.J. et al, Nature Metabolism 1, 790-810 (2019); Noormohammadi A et al, Nature Communications 7, 13649 (2016); Amrit F.R. et al, Methods 68, 465–475 (2014); Zheng Q et al, Cell 174, 870-883 (2018)).

Data exclusions

No data were excluded from the analyses.

Replication

At least three independent experiments for each assay were performed to verify the reproducibility of the findings (if there were two independent experiments, this was also noticed in the figure legend). All the attempts of replication gave a similar outcome. Lifespan assays were done at least 2 times with 96 animals per each condition. Exact numbers and statistics are provided in figure legends and supplementary data.

Randomization

For lifespan experiments, imaging experiments, and motility assays, worms were synchronized by picking young hermaphrodites adults and let them lay eggs for 6 hours. These young hermaphrodites were randomly picked from our maintenance plates. After egg laying for 6 hours, larvae were raised until adulthood and adult worms were then randomly assigned to the different treatment conditions. The different conditions were assessed in random order.
For proteomics, filter trap assays and western blot experiments, worms were synchronized by bleaching of young hermaphordite worms followed by L1 starvation standard procedures. These young hermaphrodite worms were obtained by transferring random chunks of agar from maintenance plates and let them grow until we have sufficient young hermaphrodites worms for bleaching. After bleaching worms and obtaining synchronized adults, these young worms were randomly assigned to the different treatment conditions. Then, the samples were collected, lysed and analyzed in random order.

Blinding

For proteomics experiments, sample collection was not performed in a blinded manner as aged wild-type worms, long-lived mutants and RNAi-treated worms have obvious phenotypes that revealed the sample identity (i.e., ages, strains and treatments). Once the samples were processed for proteomics, the mass spectrometry was performed by the staff of the CECAD Proteomics Facility in a blinded manner. Blinded analysis of proteomics data was not feasible as it required integrative analysis of different conditions.

qPCR, filter trap and western blot experiments were not performed in a blinded manner as they rely on objective instrument measurements and/or provide indirect outputs.
For lifespan, microscopy, motility and bacterial colonization, the experiments were not performed in a blinded manner given the nature of the reagents used (e.g., HT115 E. coli carrying empty vector or RNAi clones have to be refreshed every day during early adulthood and very other day at later stages). However, worms were randomly assigned to the different treatment conditions and the different conditions were assessed in random order. Moreover, all the critical experiments were repeated independently by at least 2 of the investigators.

The investigators were not blinded during data analysis due to feasibility of the analysis.

Reporting for specific materials, systems and methods

We require information from authors about some types of materials, experimental systems and methods used in many studies. Here, indicate whether each material, system or method listed is relevant to your study. If you are not sure if a list item applies to your research, read the appropriate section before selecting a response.

Materials & experimental systems

n/a	Involved in the study
☐	☒ Antibodies
☒	☐ Eukaryotic cell lines
☒	☐ Palaeontology and archaeology
☐	☒ Animals and other organisms
☒	☐ Human research participants
☒	☐ Clinical data
☒	☐ Dual use research of concern

Methods

n/a	Involved in the study
☒	☐ ChIP-seq
☒	☐ Flow cytometry
☒	☐ MRI-based neuroimaging

Antibodies

Antibodies used

We used the following antibodies in this study:
anti-IFB-2 (Developmental Studies Hybridoma Bank, MH33, 1:1,000. RRID: AB_528311).
anti-EPS8L2 (Abcam, ab85960, 1:1,000. RRID: AB_1924963)
anti-α-tubulin (Sigma, T6199, 1:5,000. RRID: AB_477583)
anti-JNK (Cell Signaling, #9252, 1:1,000. RRID: AB_2250373)
anti-Phospho-JNK (Thr183/Tyr185) (Cell Signaling, #9251, 1:1,000. RRID: AB_331659)
anti-ubiquitin (Sigma, #05-944, Clone P4D1-A11, 1:1,000. RRID: AB_441944)
anti-GFP (AMSBIO, 210-PS-1GFP, 1:5,000. RRID: AB_10013682)
anti-β-actin (Abcam, ab8226, 1:5,000. RRID: AB_306371)
anti-Ubiquitin Antibody, Lys48-Specific, clone Apu2 (Merck, #05-1307, 1:50. RRID: AB_1587578)
anti-Ubiquitin Antibody, Lys63-Specific, clone Apu3 (Merck, #05-1308, 1:50. RRID: AB_1587580)
anti-FLAG antibody (SIGMA, F7425, 1:100. RRID: AB_439687)

Validation

Validation of antibodies were done by the stated manufacturer's and supported by the publications indicated in the manufacturer's website, the Resource Identification Portal (RRID) and other publications using C. elegans as a model organism (including our previous publications).

* anti-IFB-2 (Developmental Studies Hybridoma Bank, MH33, 1:1,000. RRID: AB_528311). The antibody was used according to the manufacturer's instructions for western blot in C. elegans. References: PMID:31414984, PMID:31414984.

* anti-EPS8L2 (Abcam, ab85960, 1:1,000. RRID: AB_1924963) was validated in C. elegans by the data presented in this manuscript (1- the increased in EPS-8 levels observed by proteomics during aging correlated with increased levels by western blot, 2- ubiquitin-less EPS-8 protein cannot be degraded by the proteasome and accordingly western blot shows increase levels of EPS-8 in ubiquitin-less EPS-8 mutant strain).

* anti-α-tubulin (Sigma, T6199, 1:5,000. RRID: AB_477583). The antibody was validated as a loading control for western blot analysis in C. elegans in our previous publications: PMID: 32451438, PMID: 27892468.

* anti-JNK (Cell Signaling, #9252, 1:1,000. RRID: AB_2250373). According to the manufacturer and supported by previous studies, this antibody has been validated for western blot in multiple species including C. elegans (PMID: 15767565, PMID:23525221, PMID:23715867, PMID:24773344, PMID:25209287, PMID:25849727, PMID:26153447, PMID:26295369, PMID:26881311, PMID:27145004, PMID:27253999).

* anti-Phospho-JNK (Thr183/Tyr185) (Cell Signaling, #9251, 1:1,000. RRID: AB_331659). According to the manufacturer and supported by published studies, this antibody has been validated for western blot in multiple species including C. elegans (PMID: 15767565, PMID:24635351, PMID:24773344, PMID:25164676, PMID:25209287, PMID:25849727, PMID:25885794, PMID:26132918, PMID:26153447, PMID:27145004, PMID:27253999).

*anti-ubiquitin (Sigma, #05-944, Clone P4D1-A11, 1:1,000. RRID: AB_441944). Since ubiquitin is evolutionary conserved among species, this antibody is predicted to work on a wide range of species. Accordingly, it has been validated for western blot application in multiple species (PMID:27552055, PMID:29499138, PMID:29547723). Its application for western blot in C. elegans has been validated in the experiments presented in this manuscript. For instance, it can be used to detect ubiquitinated proteins as demonstrated in the western blots presented in Figs. 1g-i where global changes in ubiquitination correlated with the results observed by ubiquitin proteomics Moreover, we could reverse loss of Ub-protein levels detected by western blot by adding broad-spectrum deubiquitinase inhibitor (Fig. 1j).

* anti-GFP (AMSBIO, 210-PS-1GFP, 1:5,000. RRID: AB_10013682). This antibody has been validated for filter trap and western blot in C. elegans in our previous publications: PMID: 27892468; PMID: 30038412

* anti-β-actin (Abcam, ab8226, 1:5,000. RRID: AB_306371). This antibody has been validated for use in C. elegans in our previous publication PMID: 30038412

* anti-Ubiquitin Antibody, Lys48-Specific, clone Apu2 (Merck, #05-1307, 1:50. RRID: AB_1587578). Since Lys48-linked polyubiquitin chains are evolutionary conserved among species, this antibody is predicted to work on a wide range of species. Accordingly, it has been validated for western blot and immunoprecipitation application in multiple species (PMID:27523608, PMID:28347402, PMID:28594325, PMID:28712572, PMID:28943312, PMID:29024643, PMID:29153505, PMID:30581143, PMID:31042464, PMID:31613024, PMID:31825842). We have validated its use for immunoprecipitation experiments in C. elegans in this manuscript by confirming that age-dysregulated proteasome targets contain Lys48-linked polyubiquitin chains (Fig. 2b).

* anti-Ubiquitin Antibody, Lys63-Specific, clone Apu3 (Merck, #05-1308, 1:50. RRID: AB_1587580). Since Lys63-linked polyubiquitin chains are evolutionary conserved among species, this antibody is predicted to work on a wide range of species. Accordingly, it has been validated for western blot and immunoprecipitation application in multiple species (PMID:27523608, PMID:28244869, PMID:28594325, PMID:28712572, PMID:28943312, PMID:29153505, PMID:29547723, PMID:29576527, PMID:29861391, PMID:30893611, PMID:30901564, PMID:31042464, PMID:31606272, PMID:31613024, PMID:31825842). We have validated its use for immunoprecipitation experiments in C. elegans in this manuscript by confirming that most of the age-dysregulated proteasome targets do not contain Lys63-linked polyubiquitin chains (Extended Data Fig. 4d).

* anti-FLAG antibody (SIGMA, F7425, 1:100. RRID: AB_439687). We have validated the use of anti-FLAG antibody as a negative control for immunopreciptation experiments followed by label-free proteomics in our previous publications (PMID: 32451438, PMID: 30038412)

Animals and other organisms

Policy information about studies involving animals; ARRIVE guidelines recommended for reporting animal research

Laboratory animals

Caenorhabditis elegans strains were used in this study. For all the experiments, we used hermaphrodites worms. For label-free quantitative proteomics, wild-type, daf-2(e1370) and eat-2(ad1116) worms were collected with M9 buffer at day 1, 5, 10 and 15 of adulthood. For proteomics analysis of proteasome-less worms, we collected day 5 adult worms treated with either Vector RNAi or rpn-6 RNAi. Lifespan analysis was started from day 1 of adulthood. For all the other experiments, the specific age is indicated in the corresponding figures and/or figure legends.

The C. elegans strains used in this study are:

Wild-type (N2)
DA1116 (eat-2 (ad1116)II)
RW1596 (myo-3(st386)V; stEx30[myo-3p::GFP:: myo-3 + rol-6(su1006)])
CF1041 (daf-2(e1370)III)
BJ49 (kcIs6[ifb-2p::ifb-2a::CFP]IV)
BJ186 (kcIs30[ifo-1p::ifo-1::YFP;myo-3p::mCherry::unc-54]III)
BJ324 (kcEx78[ifc-1p::ifc-1::eGFP; unc- 119(ed3)+];unc-119(ed3)III)
BJ316 (ifc-2(kc16[ifc-2a/e::YFP])X)
BJ312 (kcIs40[ifp-1p::ifp-1::eGFP]IV)
AGD1657 (unc-119(ed3)III; uthSi13[gly-19p::LifeAct::mRuby::unc-54 3'UTR::cb-unc-119(+)]IV)
AGD1654 (unc-119(ed3)III; uthSi10[col-19p::LifeAct::mRuby::unc-54 3'UTR::cb-unc-119(+)]IV)
DVG197 (N2, ocbEx162[sur-5p::ifb-2, myo-3p::GFP])
DVG198 (N2, ocbEx163[sur-5p::ifb-2, myo-3p::GFP])
DVG9 (N2, ocbEx9[myo3p::GFP])
VP303 (rde-1(ne219)V; kbIs7[nhx-2p::rde-1 + rol-6(su1006)])
WM118 (rde-1(ne300)V; neIs9[myo-3p::HA::RDE-1 + rol-6(su1006)]
TU3401 (sid-1(pk3321)V; uIs69[pCFJ90(myo-2p::mCherry)+unc-119p::sid-1])
NR222 (rde-1(ne219)V; kzIs9 [(pKK1260) lin-26p::NLS::GFP + (pKK1253) lin-26p::rde-1 + rol-6(su1006)])
VDL07 (ifb-2(syb2876)II)
VDL05 (eps-8(syb2901)IV)
VDL06 (eps-8(syb2901, syb3149)IV)
VDL08 (ifb-2(syb3973)II))

Wild animals

The study did not involve wild animals.

Field-collected samples

The study did not involve samples collected from the field.

Ethics oversight

In this research, we used invertebrate C. elegans as an organismal model and no ethical approval was required. According to the “Zentrale Kommission für die Biologische Sicherheit” (ZKBS), the responsible entity inside the Bundesamt für Verbraucherschutz und Lebensmittelsicherheit to assess the risk of Genetically Modified Organisms (GMO), genetic work with C. elegans is classified as risk group 1 (biological safety level 1: S1). Accordingly, we performed work on C. elegans in a S1-laboratory. The use of GMO in Germany

is regulated by the “Gentechnik-Gesetz”, and we followed the guidelines applying to S1 work with GMO (i.e., documentation of the project and of the, exact description of the creation and maintenance of the genetic modification or correct waste treatment).

Note that full information on the approval of the study protocol must also be provided in the manuscript.
